# Retraction: miR-181a-5p Promotes Proliferation and Invasion and Inhibits Apoptosis of Cervical Cancer Cells via Regulating Inositol Polyphosphate-5-Phosphatase A (INPP5A)

**DOI:** 10.32604/or.2024.056126

**Published:** 2024-08-23

**Authors:** Oncology Research Editorial Office

The published article titled “miR-181a-5p Promotes Proliferation and Invasion and Inhibits Apoptosis of Cervical Cancer Cells via Regulating Inositol Polyphosphate-5-Phosphatase A (INPP5A)” has been retracted from *Oncology Research*, Vol. 26, No. 5, 2018, pp. 703–712.

DOI: 10.3727/096504017X14982569377511

URL: https://www.techscience.com/or/v26n5/56683

Following the publication, concerns have been raised about a number of figures in this article. An unexpected area of similarity was identified in terms of the cellular data, where the results from differently performed experiments were intended to have been shown, although the areas immediately surrounding this area featured comparatively different distributions of cells. In addition, the western blots in this article were presented with atypical, unusually shaped and possibly anomalous protein bands in many cases.

The authors were contacted and invited to comment on the concerns raised and to provide the original, unmodified figures, but did not respond. The Editors-in-Chief therefore no longer have confidence in the integrity of the data in this article and decided to retract this article.

All authors have not responded to correspondence about this retraction. As a responsible publisher, we hold the reliability and integrity of our published content in high regard. We deeply regret any inconvenience caused by this situation to our readers and all concerned parties.

